# Continuous endotracheal tube cuff pressure control system protects against ventilator-associated pneumonia

**DOI:** 10.1186/cc13837

**Published:** 2014-04-21

**Authors:** Leonardo Lorente, María Lecuona, Alejandro Jiménez, Lisset Lorenzo, Isabel Roca, Judith Cabrera, Celina Llanos, María L Mora

**Affiliations:** 1 Department of Critical Care, Hospital Universitario de Canarias, Ofra s/n, La Cuesta, La Laguna 38320, Santa Cruz de Tenerife, Spain; 2 Department of Microbiology and Infection Control, Hospital Universitario de Canarias, Ofra s/n, La Cuesta, La Laguna 38320, Santa Cruz de Tenerife, Spain; 3 Research Unit, Hospital Universitario de Canarias, Ofra s/n, La Cuesta, La Laguna 38320, Santa Cruz de Tenerife, Spain

## Abstract

**Introduction:**

The use of a system for continuous control of endotracheal tube cuff pressure reduced the incidence of ventilator-associated pneumonia (VAP) in one randomized controlled trial (RCT) with 112 patients but not in another RCT with 142 patients. In several guidelines on the prevention of VAP, the use of a system for continuous or intermittent control of endotracheal cuff pressure is not reviewed. The objective of this study was to compare the incidence of VAP in a large sample of patients (n = 284) treated with either continuous or intermittent control of endotracheal tube cuff pressure.

**Methods:**

We performed a prospective observational study of patients undergoing mechanical ventilation during more than 48 hours in an intensive care unit (ICU) using either continuous or intermittent endotracheal tube cuff pressure control. Multivariate logistic regression analysis (MLRA) and Cox proportional hazard regression analysis were used to predict VAP. The magnitude of the effect was expressed as odds ratio (OR) or hazard ratio (HR), respectively, and 95% confidence interval (CI).

**Results:**

We found a lower incidence of VAP with the continuous (n = 150) than with the intermittent (n = 134) pressure control system (22.0% versus 11.2%; p = 0.02). MLRA showed that the continuous pressure control system (OR = 0.45; 95% CI = 0.22-0.89; p = 0.02) and the use of an endotracheal tube incorporating a lumen for subglottic secretion drainage (SSD) (OR = 0.39; 95% CI = 0.19-0.84; p = 0.02) were protective factors against VAP. Cox regression analysis showed that the continuous pressure control system (HR = 0.45; 95% CI = 0.24-0.84; p = 0.01) and the use of an endotracheal tube incorporating a lumen for SSD (HR = 0.29; 95% CI = 0.15-0.56; p < 0.001) were protective factors against VAP. However, the interaction between type of endotracheal cuff pressure control system (continuous or intermittent) and endotracheal tube (with or without SSD) was not statistically significant in MLRA (OR = 0.41; 95% CI = 0.07-2.37; p = 0.32) or in Cox analysis (HR = 0.35; 95% CI = 0.06-1.84; p = 0.21).

**Conclusions:**

The use of a continuous endotracheal cuff pressure control system and/or an endotracheal tube with a lumen for SSD could help to prevent VAP in patients requiring more than 48 hours of mechanical ventilation.

## Introduction

Ventilator-associated pneumonia (VAP) continues to be an important cause of morbidity and mortality in critically ill patients [1-6].

Tracheal tube cuff-pressure should be sufficiently high to prevent leaks that could make mechanical ventilation ineffective, and to prevent the progression of secretions from the oropharynx towards the lower airway, in order to reduce the appearance of VAP. In one study, patients with persistently low tracheal-tube cuff pressure below 20 cm H_2_O showed a higher incidence of VAP [7]. In this study, including 83 intubated patients undergoing continuous subglottic secretion drainage (SSD), the authors found that patients with persistent intra-cuff pressures below 20 cm H_2_O showed a trend towards a higher risk of VAP (relative risk (RR) = 2.57, 95% CI = 0.78, 8.03), and a statistically significant risk of VAP among patients not receiving antibiotics (RR = 4.23, 95% CI = 1.12, 15.92). On the other hand, tracheal-tube cuff pressure should not exceed 30 cm H2O to avoid vascular compromise of the trachea, which could result in tracheomalacia and even tracheal necrosis [8,9].

A preventive strategy to avoid the progression of subglottic secretions into the lower respiratory tract is the use of a system for continuous control of endotracheal-tube cuff pressure. In one randomized controlled trial (RCT) published in 2007, which included 142 mechanically ventilated patients, there were no significant differences in the incidence of VAP between groups treated with a continuous or an intermittent endotracheal-tube cuff-pressure control system [10]. However, another RCT published in 2011, with 122 patients expected to receive mechanical ventilation for at least 48 hours, found a lower incidence of VAP with the use of a continuous compared to an intermittent endotracheal-tube cuff-pressure control system [11].

In several guidelines on the prevention of VAP, the issue of endotracheal-tube cuff-pressure control is not reviewed [12-15]. Other guidelines only recommend maintaining optimal tube cuff-pressure but make no recommendations on the use of a continuous or intermittent tube cuff-pressure control system [16-19].

Thus, the objective of this research was to compare the incidence of VAP in a large sample of mechanically ventilated ICU patients receiving either a continuous or an intermittent endotracheal-tube cuff-pressure control system. We hypothesized that the use of a continuous endotracheal cuff-pressure control system could help to prevent VAP. We would have preferred to use an endotracheal tube with a lumen for SSD in all patients as several RCTs have shown that this reduced the incidence of VAP [20-22], and furthermore, it has been recommended in several guidelines [12-15,17-19]. However, due to financial constraints, endotracheal tubes without a lumen for SSD were used in some patients; precisely because of this limitation, we were able to analyze the impact of continuous control of cuff pressure and tube with a lumen for SSD on the incidence of VAP.

## Methods

### Design of the study

A prospective observational study with an incidental sample of 284 patients was performed at the 24-bed medical-surgical ICU of the Hospital Universitario de Canarias (Tenerife, Spain), a 650-bed tertiary hospital, during one year. The study was approved by the Institutional Review Board of the Hospital Universitario de Canarias (Tenerife, Spain). Informed consent was obtained from the patients or their legal guardians. The inclusion and exclusion criteria were as follows: inclusion - patients requiring mechanical ventilation; exclusion - patients requiring mechanical ventilation for less than 48 hours (as their risk of VAP is low).

### VAP prevention measures

Patients who were admitted to an odd-numbered ICU cubicle received a continuous cuff-pressure system (Mallinckrodt Pressure Control®; VBM Medizintechnik GmbH, Sulz am Neckar, Germany) and those admitted to an even-numbered ICU cubicle received an intermittent cuff-pressure system (Mallinckrodt Pressure Manometer®; Mallinckrodt, Athlone, Ireland). In both patient groups, intra-cuff pressure was verified every 8 hours to maintain it at 25 cm H_2_O and pressure values were recorded in the chart of each patient. Each type of cuff-pressure system was applied from the beginning of connection to mechanical ventilation.

Endotracheal tubes used were Mallinckrodt™ TaperGuard Evac Oral Tracheal Tube (Covidien, Mansfield, MA, USA) which incorporates a taper-shaped cuff of polyvinylchloride (PVC) and a lumen for SSD, which was performed intermittently during 1-hour periods with a 10-mL syringe; and Mallinckrodt™ Hi-Lo Tracheal Tube (Mallinckrodt,) with a cylindrical-shaped cuff of PVC and without a lumen for SSD.

No routine change of ventilator circuits was performed. Tracheal suction when necessary was performed using an open system and with strict barrier measures before airway management (hand washing, use of gloves and face masks).

Oral cleansing was performed by nurses every 8 hours as follows: first, the endotracheal cuff-pressure was tested and oropharyngeal secretions were aspirated, then gauze-impregnated with 20 mL of 0.12% chlorhexidine digluconate, which was used to cleanse the teeth, tongue, and mucosal surfaces, followed by the injection of 10 mL of 0.12% chlorhexidine digluconate into the oral cavity, and, finally, after 30 seconds, the oropharyngeal area was suctioned.

Semi-recumbent body position to maintain an angle of 40° was verified every 4 hours. Residual gastric volume was verified every 6 hours (residual gastric volume lower than 250 cc was considered acceptable). No selective digestive decontamination was performed. Short-course (2 days) systemic antibiotic therapy was administered to patients with a decreased level of consciousness at the time of intubation. The sedation drugs were adjusted to achieve a level of 3 to 4 on the Ramsay scale [23].

### Microbiological vigilance

Tracheal aspirate samples were obtained during endotracheal intubation, then twice a week and finally on extubation. Throat swabs were taken on admission to ICU, then twice a week and at discharge from the unit.

### Definitions

The diagnosis of pneumonia was established, as in a previous study by our team [24], when all of the following criteria were met: a) new onset of bronchial purulent sputum; b) body temperature >38°C or <35.5°C; c) white blood cell count >10,000/mm^3^ or <4,000/mm^3^; d) chest radiograph showing new or progressive infiltrates; and e) significant quantitative culture of respiratory secretions by tracheal aspirate (>10^6^ cfu/mL). The criteria for diagnosis of tracheobronchitis were the same as those for pneumonia, but without change demonstrated on the chest radiograph.

Pneumonia was considered as VAP when it was diagnosed after 48 hours of mechanical ventilation. The diagnosis of VAP was made by an expert panel blinded to cuff-pressure system. VAP was considered as early onset when it was diagnosed during the first 4 days of mechanical ventilation. VAP was considered as late onset when it was diagnosed after 4 days of mechanical ventilation.

VAP was classified pathogenically, according to throat flora, as primary endogenous, secondary endogenous or exogenous [25]. VAP was considered as primary endogenous when caused by microorganisms already present in the patient’s oropharyngeal flora on admission to ICU. VAP was considered as secondary endogenous when caused by microorganisms not found on admission but detected in the patient’s oropharyngeal flora during the ICU stay. VAP was considered as exogenous when it was caused by microorganisms that were never carried in the patient’s oropharyngeal flora.

### Variables recorded

The following variables were recorded for each patient: sex; age; diagnostic group; type of admission; smoking status; chronic obstructive pulmonary disease; diabetes mellitus; use of chemotherapeutic agents or steroid agents; hematological tumor; solid tumor; diagnosis group; Acute Physiology and Chronic Health Evaluation (APACHE)-II score [26]; duration of mechanical ventilation; antibiotics prior to VAP onset; use of paralytic agents; tracheotomy; reintubation; enteral nutrition; type of endotracheal-tube cuff-pressure control system (continuous or intermittent); type of endotracheal tube (with or without a small-bore lumen for SSD); positive end-expiratory pressure (PEEP); Ramsay scale [23]; head-of-bed angle elevation; red blood cell transfusion; cuff pressure; and ICU mortality.

### Statistical analysis

Quantitative variables are reported as mean ± SD, and were compared using the Student *t*-test. Qualitative variables are reported as frequency and percentage, and were compared using the Chi-squared test and (in the case of small samples) the Fisher exact test. We used the Kruskal-Wallis test for singly ordered row (R) x column (C) tables to compare proportions of patients who received the intermittent/continuous cuff-pressure control system in the diagnostic group, type of admission and stress-ulcer prophylaxis. The probability of remaining VAP-free was represented using the Kaplan-Meier method and comparison between the two groups was performed with the log-rank test.

Multivariate logistic regression analysis (MLRA) was used to assess the risk of VAP and the variables included were: type of endotracheal-tube cuff-pressure control system (continuous or intermittent); type of endotracheal tube (with or without a small-bore lumen for SSD); APACHE-II score; use of paralytic agents; reintubation; enteral nutrition; duration of mechanical ventilation; and the interaction between the type of endotracheal-tube cuff-pressure control system (continuous or intermittent) and endotracheal tube (with or without SSD). The magnitude of the effect was expressed as the odds ratio (OR) and 95% CI.

Cox proportional hazard regression analysis using the step-by-step method to select the predictor variables was used, with VAP-free time as the dependent variable, VAP as the event, and the type of endotracheal-tube cuff-pressure control system (continuous or intermittent) as the main independent variable, and controlling for the APACHE-II score, use of paralytic agents, reintubation, enteral nutrition, type of endotracheal tube (with or without a small-bore lumen for subglottic secretion drainage) and the interaction between type of endotracheal cuff-pressure control system (continuous or intermittent) and endotracheal tube (with or without subglottic secretion drainage). The magnitude of the effect was expressed as the hazard ratio (HR) and 95% CI. In both regression analyses we introduced those variables found to be associated with the risk of VAP [27]. As the number of patients with VAP was 48, we only included seven variables in the regression analyses to avoid an over-fitting effect. A *P*-value <0.05 was considered statistically significant. For statistical analyses, we used SPSS 17.0.1 (SPSS Inc., Chicago, IL, USA) and StatXact 5.0.3 (Cyrus Mehta and Nitin Patel, Cambridge, MA, USA).

## Results

There were no significant differences between the two groups of patients (150 with the intermittent and 134 with continuous cuff-pressure control system) in terms of sex, age, smoking status, chronic obstructive pulmonary disease, diabetes mellitus, chemotherapeutic agents, steroid agents, hematological tumor, solid tumor, diagnosis group, APACHE-II score, duration of mechanical ventilation, antibiotics prior to VAP onset, use of paralytic agents, tracheotomy, reintubation, enteral nutrition, SSD or ICU mortality (Table 1). However, we found a higher incidence of VAP (22.0% versus 11.2%; *P* = 0.02) and a higher percentage of pressure-cuff determinations <20 cm H_2_0 (9.32 ± 8.46 versus 0.00; *P* < 0.001) in the group of patients with the intermittent compared to the continuous cuff-pressure control system. Compliance with the cuff-pressure control system was 100% in both patient groups and there were no cases of crossover.

**Table 1:** Characteristics of intermittent and continuous endotracheal-tube cuff-pressure control system patient groups

	**Intermittent cuff-pressure**	**Continuous cuff-pressure**	***P*-value**
	**control system (n = 150)**	**control system (n = 134)**	
Sex, female, n (%)	63 (42.0)	46 (34.3)	0.22
Age, years, mean ± SD	63.21 ± 14.99	59.61 ± 17.22	0.06
Diagnostic group, n (%)			0.81
Cardiac surgery	26 (17.3)	20 (14.9)	
Cardiology	13 (8.7)	16 (11.9)	
Respiratory	37 (24.7)	30 (22.4)	
Digestive	23 (15.3)	20 (14.9)	
Neurologic	26 (17.3)	21 (15.7)	
Trauma	17 (11.3)	22 (16.4)	
Others	8 (5.3)	5 (3.7)	
Type of admission, n (%)			0.44
Postoperative	41 (27.3)	40 (29.9)	
Medical	91 (60.7)	72 (53.7)	
Traumatic	18 (12.0)	22 (16.4)	
Smoker, n (%)	30 (20.0)	30 (22.4)	0.66
Chronic obstructive pulmonary disease, n (%)	22 (14.7)	20 (14.9)	0.99
Diabetes mellitus, n (%)	45 (30.0)	40 (29.9)	0.99
Chemotherapeutic agents, n (%)	4 (2.7)	5 (3.7)	0.74
Steroid agents, n (%)	8 (5.3)	4 (3.0)	0.39
Hematological tumor, n (%)	6 (4.0)	5 (3.7)	0.99
Solid tumor, n (%)	16 (10.7)	18 (13.4)	0.58
APACHE-II score, mean ± SD	17.53 ± 8.88	17.57 ± 7.26	0.97
Intubation in the ICU, n (%)	48 (32.0)	42 (31.3)	0.99
Subglottic secretion drainage, n (%)	65 (43.3)	53 (39.6)	0.55
Tracheotomy, n (%)	25 (16.7)	29 (21.6)	0.29
Paralytic agents, n (%)	24 (16.0)	19 (14.2)	0.74
Enteral nutrition, n (%)	103 (68.7)	88 (65.7)	0.61
Antibiotics before VAP, n (%)	144 (96.0)	132 (98.5)	0.29
Reintubation, n (%)	16 (10.7)	16 (11.9)	0.85
Stress ulcer prophylaxis, n (%)			0.11
Proton-pump inhibitors	137 (91.3)	126 (94.0)	
Histamine H-2 blockers	5 (3.3)	0	
None	8 (5.3)	8 (6.0)	
PEEP, cm H_2_0, mean ± SD	5.25 ± 0.88	5.19 ± 0.85	0.56
Ramsay scale, mean ± SD	3.37 ± 0.83	3.45 ± 0.79	0.44
Head of bed angle elevation, degrees, mean ± SD	33.13 ± 7.22	33.54 ± 7.65	0.64
Red blood cell transfusion, n (%)	52 (34.7)	54 (40.3)	0.39
P_cuff_ determinations < 20 cm H_2_0, %, mean ± SD	9.32 ± 8.46	0	<0.001
P_cuff_ determinations 20 to 30 cm H_2_0, %, mean ± SD	86.93 ± 10.07	100	<0.001
P_cuff_ determinations >30 cm H_2_0, %, mean ± SD	3.74 ± 4.85	0	<0.001
VAP, patients, n (%)	33 (22.0)	15 (11.2)	0.02
Tracheobronchitis, patients, n (%)	10 (6.7)	5 (3.7)	0.30
VAP or tracheobronchitis, patients, n (%)	43 (28.7)	20 (14.9)	0.01
Time of MV free of VAP, days, mean ± SD	10.31 ± 10.56	12.75 ± 14.05	0.10
Duration of MV, days, mean ± SD	15.65 ± 20.78	15.21 ± 15.23	0.84
ICU mortality, patients, n (%)	55 (36.7)	51 (38.1)	0.90

APACHE, acute physiology and chronic health evaluation; PEEP, positive end-expiratory pressure; MV, mechanical ventilation; VAP, ventilator-associated pneumonia; P_cuff_, cuff pressure.

Multivariate logistic regression analysis showed that the continuous pressure control system (OR = 0.45, 95% CI = 0.22, 0.89, *P* = 0.02) and the use of an endotracheal tube incorporating a lumen for subglottic secretion drainage (OR = 0.39; 95% CI = 0.19, 0.84; *P* = 0.02) were protective factors against VAP (Table 2). However, the interaction between type of endotracheal cuff-pressure control system (continuous or intermittent) and endotracheal tube (with or without SSD) was not statistically significant (OR = 0.41, 95% CI = 0.07, 2.37; *P* = 0.32).

**Table 2:** Mutilple logistic regression analysis to predict ventilator-associated pneumonia

	**Odds ratio**	**95% CI**	***P*-value**
Continuos versus intermittent endotracheal tube cuff-pressure control system	0.45	0.22, 0.89	0.02
Endotracheal tube with versus without a lumen for subglottic secretion drainage	0.39	0.19, 0.84	0.02
APACHE-II score	0.98	0.94, 1.02	0.25
Paralytic agents	1.42	0.59, 3.41	0.43
Reintubation	2.29	0.91, 5.78	0.08
Enteral nutrition	2.17	0.98, 4.83	0.06
Days of mechanical ventilation	0.98	0.94, 1.02	0.23

APACHE, acute physiology and chronic health evaluation.

Multivariate Cox regression analysis showed that the continuous pressure control system (HR = 0.45, 95% CI = 0.24, 0.84, *P* = 0.01) and the use of an endotracheal tube incorporating a lumen for SSD (HR = 0.29, 95% CI = 0.15, -0.56, *P* <0.001) were protective factors against VAP (Table 3). However, the interaction between type of endotracheal cuff-pressure control system (continuous or intermittent) and endotracheal tube (with or without SSD) was not statistically significant (HR = 0.35, 95% CI = 0.06, 1.84, *P* = 0.21).

**Table 3:** Cox regression analysis to predict ventilator-associated pneumonia

	**Hazard ratio**	**95% CI**	***P*-value**
Continuos versus intermittent endotracheal tube cuff-pressure control system	0.45	0.24-0.84	0.01
Endotracheal tube with versus without a lumen for subglottic secretion drainage	0.29	0.15-0.56	<0.001
APACHE-II score	0.98	0.94-1.02	0.33
Paralytic agents	0.95	0.45-2.02	0.90
Reintubation	1.48	0.71-3.10	0.30
Enteral nutrition	1.41	0.69-2.90	0.35

APACHE, acute physiology and chronic health evaluation.

**Figure 1:**
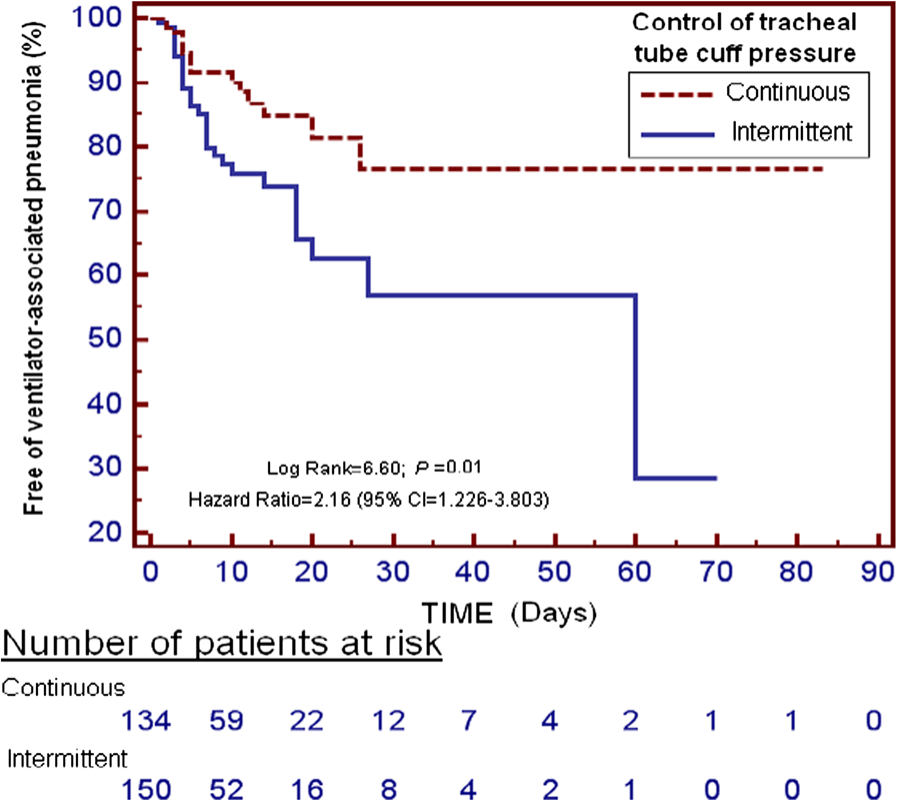
Cumulative proportion of patients remaining free of ventilator-associated pneumonia using a continuous or intermittent endotracheal tube cuff-pressure control system.

Kaplan-Meier analysis showed a lower incidence of VAP with continuous compared to intermittent cuff-pressure control system (log rank = 6.60, *P* = 0.01, HR = 2.16, 95% CI = 1.226, 3.803) (Figure 1).

Table 4 shows the microorganisms responsible for VAP according to intermittent and continuous cuff-pressure control systems. For statistical analysis, we grouped the microorganisms responsible for VAP into three groups: gram-positive bacteria, enterobacteriaceae and non-fermentative gram-negative bacteria (*Pseudomonas aeruginosa*, *Acinetobacter baumanii* and *Stenotrophomonas maltophilia*). There were no significant differences between patient groups in microorganisms responsible for VAP (*P* = 0.52). Gram-positive bacteria, enterobacteriaceae and non-fermentative gram-negative bacteria in the intermittent cuff-pressure control system group were identified in 6 (18.2%), 18 (54.5%) and 9 (27.3%) cases of VAP, respectively, versus 2 (13.3%), 10 (66.7%) and 3 (20.0%) cases of VAP, respectively, in the continuous cuff-pressure control system group.

**Table 4:** Microorganisms isolated in ventilator-associated pneumonia

**Microorganisms**	**Intermittent**	**Continuous**
	**cuff-pressure**	**cuff-pressure**
	**control system**	**control system**
TOTAL gram-positive bacteria	6	2
MSSA	4	0
MRSA	1	1
*Streptococcus pneumoniae*	1	1
TOTAL gram-negative bacteria	27	13
*Escherichia coli*	0	4
*Klebsiella spp*.	2	0
*Enterobacter spp*.	5	1
*Serratia marcescens*	5	1
*Morganella morganii*	1	0
*Proteus spp*.	0	1
*Citrobacter koseri*	2	1
*HaemophiIus influenzae*	3	2
*Pseudomonas aeruginosa*	6	3
*Stenotrophomonas maltophilia*	2	0
*Acinetobacter baumanii*	1	0
TOTAL	33	15

(MSSA, methicillin-sensitive *Staphylococcus aureus*; MRSA, methicillin-resistant *Staphylococcus aureus*).

There were no significant differences between patient groups in VAP pathogenesis (*P* = 0.73). Primary endogenous, secondary endogenous and exogenous VAP in the intermittent cuff-pressure control system group were recorded in 8 (24.2%), 24 (72.7%) and 1 (3.0%) patients, respectively, versus 2 (13.3%), 13 (86.7%) and none, respectively, in the continuous cuff-pressure control system group.

There were no significant differences between patient groups in VAP onset (*P* = 0.99). Early and late onset VAP in the intermittent cuff-pressure control system group were recorded in 13 (39.4%) and 20 (60.6%) patients, respectively, versus 6 (40.0%) and 9 (60.0%), respectively, in the continuous cuff-pressure control system group.

## Discussion

In this study, we found a significant reduction in the incidence of VAP with the use of a continuous endotracheal tube cuff pressure control system. To our knowledge, this is the largest study to date on the incidence of VAP comparing a continuous and an intermittent cuff-pressure control system.

Previously, two RCTs of small sample size analyzed the use of a continuous or an intermittent cuff-pressure control system [10,11]. In the study by Valencia *et al*., which included 142 mechanically ventilated patients, the authors found no significant differences in VAP rate between the groups (continuous versus intermittent cuff-pressure control system) (29% versus 22%; *P* = 0.44) [10]. The RCT by Nseir *et al*., including 122 patients expected to receive mechanical ventilation for at least 48 hours, found that the group of patients receiving the continuous compared to the intermittent cuff-pressure control system showed a lower rate of VAP (9.8 versus 26.2%; *P* = 0.03) [11]; however, regression analysis controlling for confounders was not reported. In our study, regression analysis showed that the use of a continuous endotracheal-tube cuff-pressure control system was associated with a significantly lower risk of VAP. The significance result on regression analysis in our study may be due to the higher sample size (n = 284) and to the patients included (patients undergoing mechanical ventilation during more than 48 hours). The benefit of the continuous in comparison to the intermittent cuff-pressure control system to reduce the risk of VAP could be due to a lower risk of deflated cuff pressure (determined by a lower percentage of determinations of cuff pressure lower than 20 cm H_2_0). Thus, more constant maintenance of cuff pressure above 20 cm H_2_0 with a continuous system could lead to a lower risk of the progression of subglottic secretions into the lower respiratory tract and finally of VAP. In addition, we found that the use of an endotracheal tube with a small-bore lumen for SSD exerted a protective effect against VAP; this finding is consistent with the results of previous studies [20-22]. As an interaction effect between SSD and the continuous endotracheal tube cuff-pressure control system was not found, we recommend both preventive measures in patients requiring more than 48 hours of mechanical ventilation to reduce the incidence of VAP and to increase VAP-free time.

Our study has certain limitations. First, the patients were not randomly assigned to receive one or the other cuff-pressure control system (continuous or intermittent). However, the method of ICU cubicle allocation introduced an element of chance: patients were admitted to the first free cubicle available. Importantly, there were no significant differences between the two groups in baseline characteristics. Second, we did not perform an assessment of pulmonary aspiration and tracheal mucosal damage. Third, the study was performed in a single ICU, and the results may therefore not be applicable to other ICUs. Fourth, regarding the blinding process, continuous or intermittent endotracheal tube cuff-pressure control systems are visually different; thus, the study could not be blinded for the attending physicians. Finally, the use of an endotracheal tube with a lumen for SSD was based on availability in the hospital due to financial constraints; thus, the tube with SSD was only used in 41.5% of all patients. However, due to this limitation, we were able to analyze the impact of the continuous control of cuff pressure and an endotracheal tube with a lumen for SSD on the incidence of VAP.

However, our study also has certain strengths. First, this is the largest study to date on the incidence of VAP comparing endotracheal-tube cuff-pressure control systems (continuous versus intermittent), including 284 patients in comparison to 122 [11] and 142 patients [10] in previous studies. Second, we found a lower incidence of VAP with the use of a continuous cuff-pressure control system in the MLRA, not only in the Kaplan-Meier analysis as in the study by Nseir *et al*. [11]. Third, the type of system for cuff-pressure control (continuous or intermittent) and the type of endotracheal tube (with or without a lumen for SSD) were blinded for the expert panel that established the diagnosis of VAP. Lastly, microbiological vigilance was based on tracheal aspirate and throat swabs twice a week and we found no differences in the pathogenesis or the microorganisms responsible for VAP between the groups receiving either continuous or intermittent cuff-pressure control.

Current guidelines on the prevention of VAP do not contain recommendations for the use of a continuous or an intermittent endotracheal-tube cuff-pressure control system [12-19]. Thus, despite the limitations of our study, our findings could help in decision-making on VAP prevention measures. They support the use of a continuous endotracheal-tube cuff-pressure control system.

## Conclusion

The use of a continuous endotracheal cuff-pressure control system and/or an endotracheal tube with a lumen for SSD could help to prevent VAP in patients requiring more than 48 hours of mechanical ventilation.

## Key messages

•The use of a continuous endotracheal cuff-pressure control system and/or an endotracheal tube with a lumen for SSD could help to prevent VAP in patients requiring more than 48 hours of mechanical ventilation.

## Abbreviations

APACHE: acute physiology and chronic health evaluation; HR: hazard ratio; MLRA: multivariate logistic regression analysis; OR: odds ratio; PEEP: positive end-expiratory pressure; PVC: polyvinylchloride; RCT: randomized controlled trial; RR: relative risk; SSD: subglottic secretion drainage; VAP: ventilator-associated pneumonia.

## Competing interests

The authors declare that they have no competing interests.

## Author contributions

LeL was responsible for the conception, design and coordination of the study; made substantial contributions to data acquisition, analysis and interpretation, and drafted the manuscript. ML, LiL, IR, JC, CL and MLM made substantial contributions to data acquisition and provided useful suggestions. AJ made substantial contributions to analysis and interpretation of data. All authors have revised the manuscript and approved the final version of manuscript.
